# Longitudinal survey of *Staphylococcus aureus *in cystic fibrosis patients using a multiple-locus variable-number of tandem-repeats analysis method

**DOI:** 10.1186/1471-2180-10-24

**Published:** 2010-01-27

**Authors:** Hoang Vu-Thien, Katia Hormigos, Gaëlle Corbineau, Brigitte Fauroux, Harriet Corvol, Didier Moissenet, Gilles Vergnaud, Christine Pourcel

**Affiliations:** 1 Hôpital Armand Trousseau, Assistance Publique-Hôpitaux de Paris (AP-HP), Bactériologie, 75012 Paris, France; 2 Université Paris Sud 11, CNRS, UMR 8621, Institut de Génétique et Microbiologie, Orsay, 91405, France; 3 Hôpital Armand Trousseau, Assistance Publique-Hôpitaux de Paris (AP-HP), Pneumologie-Pédiatrie, 75012 Paris, France; 4 DGA/MRIS- Mission pour la Recherche et l'Innovation Scientifique, 92220 Bagneux, France

## Abstract

**Background:**

*Staphylococcus aureus *infection in patients with cystic fibrosis (CF) is frequent and may be due to colonization by a few pathogenic lineages. Systematic genotyping of all isolates, methicillin-susceptible *S. aureus *(MSSA) as well as methicillin-resistant *S. aureus *(MRSA) is necessary to identify such lineages and follow their evolution in patients. Multiple-locus variable-number tandem repeat analysis (MLVA/VNTR) was used to survey *S. aureus *clinical isolates in a French paediatric CF centre.

**Results:**

During a 30 months period, 108 patients, aged 2 to 21 years, regularly followed up at the centre, provided sputum for culture. From 79 patients, a total of 278 isolates were genotyped by MLVA, resolving into 110 genotypes and 19 clonal complexes (CC) composed of similar or closely related isolates. 71% of the strains were distributed into four main CCs, in term of number of isolates and number of genotypes. *Spa *(*Staphylococcus *protein A) typing was performed on representative samples, showing an excellent concordance with MLVA. In 17 patients, strains from two to four different CCs were recovered over time. On six occasions, *S. aureus *isolates with the same genotype were shared by 2 different patients and they belonged to one of the four main clusters. Methicillin-resistance was observed in 60% of the isolates, 90% of which belonged to the main clonal complexes CC8, CC45 and CC5. In 5 patients, methicillin-resistance of *S. aureus *isolates was not associated with the *mecA *gene: for four patients, it was due to overproduction of β-lactamase, leading to BOR-SA (borderline *S. aureus*) isolates, while a strain showing probably a new modified penicillin-binding capacity (MOD-SA) was observed from one patient.

**Conclusion:**

Systematic genotyping of *S. aureus *isolates recovered from sputum of CF children allows a thorough analysis of the strains responsible for sporadic as well as chronic colonization and the follow up of their evolution over time. We show here that more than 70% of these strains belong to 4 major CCs. MSSA as well as MRSA, BOR-SA and MOD-SA isolates can persist over several years, despite antibiotic treatments.

## Background

Cystic fibrosis (CF) is caused by a mutation in the CFTR-gene leading to dysfunction of the exocrine glands. The disease is responsible for chronic airway obstruction in the lung, a favourable condition for pulmonary infections during childhood. In different studies investigating pathogens in CF, *S. aureus *was observed in 4 to 60% of patients frequently in association with other bacteria, such as *Pseudomonas aeruginosa *[1-3]. Since the introduction of methicillin in 1959, methicillin-resistant *S. aureus *(MRSA) clones have rapidly emerged and spread worldwide and account for 10 to 30% of *S. aureus *infections [4,5]. Molecular epidemiology studies using Multi Locus Sequence Typing (MLST) on clinical strains of *S. aureus *have shown that they are distributed into 11 major clonal complexes (CC) [6]. MRSA strains represent the most threatening challenge as they are frequently resistant to many antibiotics and there is evidence that antibiotic treatments not only facilitate the spreading of these clones but also enhance their pathogenicity [7]. Patients with CF are at particular risk for pulmonary colonization of MRSA, both because of their difficulty in clearing mucus and because of their frequent hospital visits, which can increase exposure to MRSA. Several studies reported that 20 to 35% of CF patients harbored a MRSA strain and described the emergence of community-acquired MRSA (CA-MRSA) [8-11]. Methicillin-susceptible strains (MSSA) also constitute a risk in CF patients, particularly because of the existence of biofilms in the infected lung in which they can escape from antibiotic treatment [12].

The epidemiology of *S. aureus *in CF patients has been investigated in different studies, but mostly MRSA were analysed and the role of MSSA was not assessed. In order to extend the knowledge of the population of *S. aureus *chronically infecting CF patients, all the isolates should be systematically genotyped with a high degree of discrimination which is difficult using the currently available techniques. The polymorphism of the *Staphylococcus *protein A gene (*spa*), first used by Frenay *et al*. [13] to genotype *S. aureus *and further evaluated by Shopsin *et al*. [14] has proven to be very useful to investigate *S. aureus *genetic diversity. Subsequently MLST became the most widely used technique to analyse the epidemiology of *S. aureus *and to perform phylogenetic studies [15]. Although the combined discriminatory power of *spa *typing and MLST is high, these techniques do not sufficiently discriminate within the major CCs and their cost is elevated. New approaches have been developed which use Variable Number of Tandem Repeats (VNTR) either to produce a multiple band pattern in a technique called MLVF [16,17] or to perform Multiple loci VNTR analysis (MLVA). MLVA consists in the analysis of individual VNTRs allowing the description of a strain in the form of a code easily exchangeable between laboratories [18]. MLVA with 6 VNTRs could correctly assigned isolates to outbreaks or identified isolates as unlinked [19]. Schouls *et al*. using 8 VNTRs showed that MLVA was at least as discriminatory as Pulse Field Gel electrophoresis (PFGE) and twice as discriminatory as *spa*-sequence typing [20]. Finally we recently described a very informative MLVA scheme which makes use of 14 VNTRs (MLVA-14) and demonstrated that its discriminatory power was much higher than those of MLST and *spa *typing [21].

In the present work we used the proposed MLVA-14 assay to genotype *S. aureus *isolates recovered from firstly as well as chronically colonized CF patients, over a period of 30 months. The aim of our study was to investigate the genetic diversity of MRSA and MSSA present in the sputum of CF children whether sporadically or chronically. The longitudinal survey of genotypes provided information on the variations in those strains recovered from some patients over a maximum period of 24 months.

## Results

### Clinical characteristics of *S. aureus *colonization

From a total number of 143 patients attending the Armand Trousseau CF centre during the 30 months study period, 108 provided sputum of which 79 showed one or several cultures positive for *S. aureus*. It is likely that most were community-acquired *S. aureus *contaminations as the majority of patients were outpatients. In addition there was no outbreak episode during the study period. Although this study was not designed to correlate the bacteria recovered from the sputum with the respiratory evolution of the patients, the following features may be underlined: Among the 79 patients, 38 were co-infected with *P. aeruginosa*, as observed in a previous investigation [22], making it difficult to determine the role of *S. aureus *in broncho-pulmonary exacerbations. Twenty-four of these patients harboured MSSA, 12 patients harboured MRSA and 2 patients harboured both. MRSA were mainly isolated from older patients who were treated by regular intravenous antibiotic courses, as recommended by the international guidelines. In the 45 other patients, *S. aureus *was the single species recovered from sputum cultures (sometimes with intermittent *Haemophilus influenzae *isolation); MSSA isolates were found in 34 patients, MRSA in 6 patients and both MSSA and MRSA in 5 other patients. These 45 patients were younger than those co-infected with *P. aeruginosa*. Of note, those harbouring MRSA had more respiratory exacerbations and worse lung function than those harbouring MSSA. Both methicillin-susceptible and methicillin resistant isolates were repeatedly recovered over several months.

Forty percent of patients were suffering from their first colonization with *S. aureus *while in 60% the recurrent isolation of the bacteria was indicative of colonization with exacerbations. In the later cases genotyping could show in several instances that the strain was different and therefore that several independent infections took place (see paragraph below). Patients were treated by antimicrobial drugs, however in most cases *S. aureus *was still recovered from sputum samples despite clinical improvement.

### Investigation of MRSA

In total a MRSA isolate was found at least once in 25 patients (33%) with a positive culture. Both screening techniques used here failed to detect the presence of the penicillin binding protein PBP 2a (*mec*A gene) in the resistant strains from five patients (CFU_29, CFU_41, CFU_48, CFU_51, CFU_68). Methicillin-resistance in these strains (except for patient CFU_41 strain) was caused by a high production of β-lactamase, leading to a characteristic BOR-SA (Borderline-*S. aureus*) phenotype. Isolates from patient CFU_41 did not show BOR-SA characteristics, suggesting that methicillin resistance resulted from a new modified penicillin-binding capacity (MOD-SA phenotype for Modified PBP-*S. aureus*) [23], though this modified capacity was not investigated. In patients colonized by BOR-SA or MOD-SA, isolates showing the same genotype but different susceptibility patterns were occasionally recovered from the same sputum sample, or over time in successive samples.

### The genetic diversity of strains as assessed by MLVA

A total of 278 isolates were genotyped by MLVA using fourteen VNTRs (Table 1). Overall the PCR efficiency was very satisfying and there was no difficulty in evaluating the amplicon size on 2% agarose gels. In one case, the presence of several bands with Sa0122 (*spa*) suggested the existence of two different variants of a strain in the sample. Indeed this could be confirmed by testing several colonies from a culture (data not shown).

**Table 1:** VNTRs characteristics and primers for PCR amplification [21]

VNTR^a^	repeat size (bp)	Mu50 N° repeats		oligos	Locus name
Sa0122^b^	24	10	L	AGCAGTAGTGCCGTTTGCTT	*spa*
			R	AAGACGATCCTTCAGTGAGCA	
Sa0266^c^	81	6	L	TTGGATATGAAGCGAGACCA	*coa*
			R	CTTCCGATTGTTCGATGCTT	
Sa0311	55	3	L	AGGGTTAGAGCCCGAGACAT	STAR
			R	CACGGGATTGGAACAGAAAT	
Sa0704	67	4	L	CGCGCGTGAATCTCTTTTAT	intergenic
			R	AGTCCCATATCGTGCGTTAAA	
Sa0906F	56	3	L	CATGTATTCATGGGATTGCAGC	STAR^d^
			R	CAGATTTTC CTTCAACAATTATCAC	
Sa1132	63	6	L	CGTGCATAATGGCTTACGAA	SAV1078
			R	AAGCAGCAGAAAAAGCTAAAGAA	
Sa1194	67	7	L	AGTGCAAGCGGAAATTGAAG	intergenic
			R	ATCGTGAAAAAGCCCAAAAA	
Sa1213F	56	5	L	GGCTGATGCTAAAGTTGCATTAGA	STAR
			R	GTGGCATGTTCTACAAACGTAAAC	
Sa1291	64	4	L	GGGGGAAATTCTAAGCAACC	intergenic
			R	CGAAATTTTCCACGTCGATT	
Sa1425	58	4	L	TCGTTATTAAACTACGAATTCTCGATT	STAR
			R	ATTTCGRGAATGATTCAATTCAATTTT	
Sa1729b	56	5	L	TACTTAAAAATARGAATACATAATTAG	STAR
			R	CAACAATAAATTACTTATTTGAAGTT	
Sa1866	159	3	L	CTGTTTTGCAGCGTTTGCTA	SAV1738
			R	GCAACTTGAAGAAACGGTTG	
Sa2039	56	3	L	TTCGTTCTACCCCAACTTGC	STAR
			R	GAGCCTGGGTCATAAATTCAA	
Sa1756^e^	131	1	L	AATTATAGCATATTAGAGCCCCTTA	50S ribosomal protein L27
Alias SIRU15			R	ACGTAAAGGTCGCGACAAAA	

^a ^The chromosomal position on the Mu50 genome, in kilobase-pairs is indicated in the VNTR name, for example Sa0120 is at position 120,000.

^b ^primers different from [39]

^c ^primers different from [40]

^d ^STAR stands for *S. aureus *repeat

^e ^primers different from SIRU15 [41]

**Figure 1:**
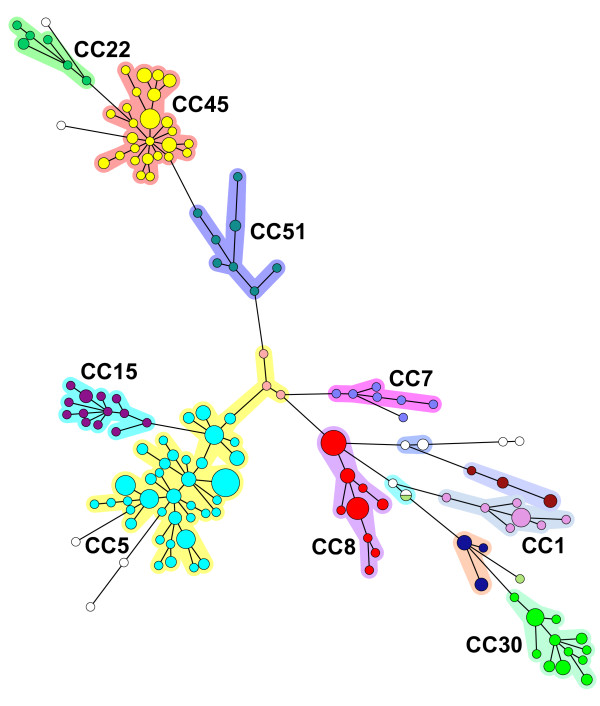
**Minimum spanning tree representation of the MLVA clustering for 278 isolates**. Each circle represents a genotype. The size is proportional to the number of samples with a given genotype (1, > = 2, > = 5, > = 10, > = 20). The corresponding MLST clonal complexes are indicated. Clusters are coloured using the same colour code as in Figure 2 and 3.

The *S. aureus *population diversity is shown in the minimum spanning tree representation on Figure 1. The presence of the 12 available sequenced genomes in the analysis allowed the identification of several MLST CCs (CC5 for Mu50, N315, JH1, JH9 reference strains and 25 patient strains, CC8 for COL, Newman, USA300, NCTC8325 reference strains and 7 patient strains, CC30 for MRSA252 strain and 6 patient strains, CC1 for MSSA476, MW2 strains and 4 patient strains). MLST was performed on two strains (TrSa176 and TrSa246) of the second largest cluster, showing that it belonged to CC45 (13 patients). A comparison made with a collection of 200 strains previously analysed by MLST and by MLVA-14 [21] confirmed the concordance of the CCs defined by the two techniques (not shown). Therefore, in the present study it was decided to use the MLST nomenclature to designate the largest CCs.

**Figure 2:**
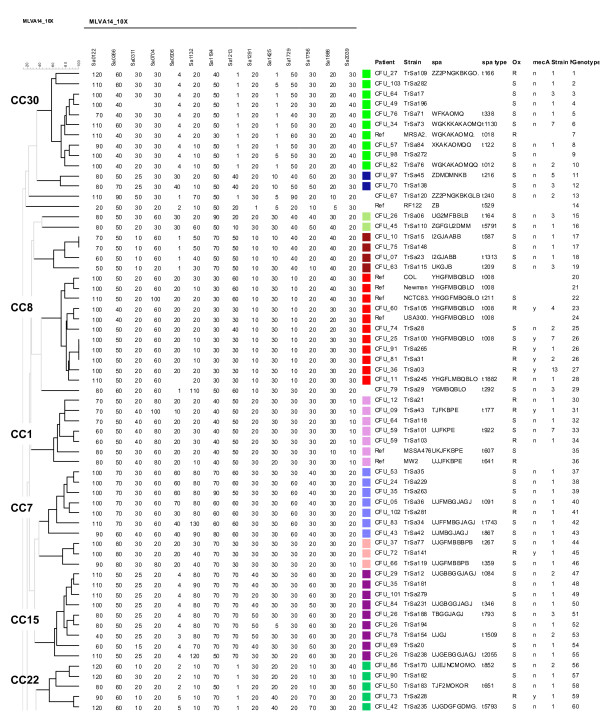
**Clustering analysis of MLVA data for 55 selected isolates and 8 reference strains**. All the CC30, CC8, CC1, CC7, CC15 and CC22 isolates cluster in this first part (genotype 1 to 60) of a dendrogram constructed from MLVA-14 testing of 116 isolates and 12 reference strains. One isolate of a given genotype was selected for each patient to produce the dendrogram (consequently some patients are represented by more than one strain). On the right are shown the patient code, the name of the selected isolate, the *spa *repeat code, the *spa *type, the methicillin (oxacillin) resistance status, the presence (y) or absence (n) of *mecA*, the number of isolates of identical genotype, the genotype number. The names of MLST clonal complexes are indicated on the left. Each cluster of two or more isolates is shown with a different coloured square using the same colour code as in Figure 1.

**Figure 3:**
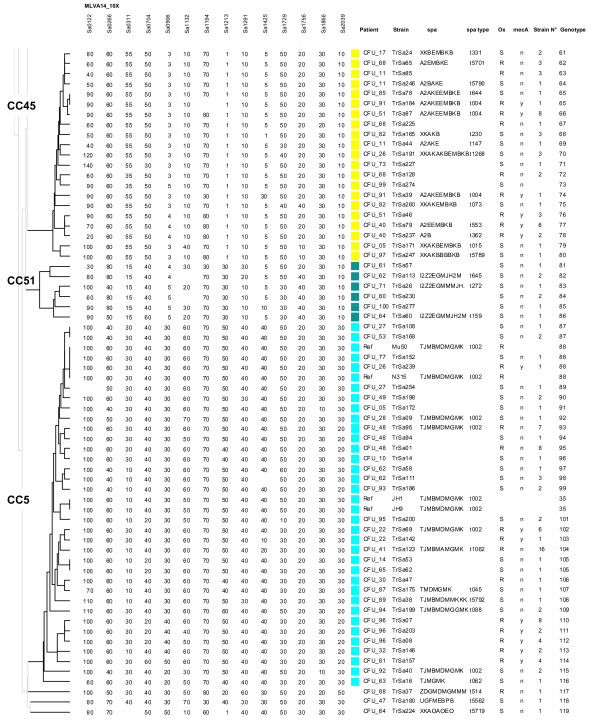
**Clustering analysis of MLVA data for 62 selected isolates and 4 reference strains**. All CC45, CC51 and CC5 isolates cluster into the second part (genotypes 61 to 119) of the dendogram constructed from MLVA-14 testing of 116 strains and 12 reference strains. One strain per genotype and per patient is included (consequently some patients are represented by more than one strain). The patient code, the name of the selected isolate, the *spa *repeat code, the *spa *type, the methicillin (oxacillin) resistance status, the presence (y) or absence (n) of *mecA*, the number of isolates of identical genotype, the genotype number are shown on the right. On the left are indicated the names of MLST clonal complexes. A different coloured square is used to indicate clusters of two or more isolates, using the same colour code as in Figure 1.

To facilitate the comparison of isolates, one strain of a given genotype per patient (117 strains and 110 genotypes) and 12 reference strains were used to perform a clustering analysis. With a cut-off value of 45% (corresponding to a maximum of three allelic differences out of 14 markers) 19 clusters, or clonal complexes (CC), were observed. Figure 2 shows the first part of a dendrogram in which all the strains belonging to CC30, CC8, CC1, CC7, CC15 and CC22 fall. The second part of the dendrogram shown on figure 3 displays all the CC45, CC51 and CC5 isolates. Four CCs comprised 71% (in term of number of isolates and number of genotypes) of the strains (CC5 35%, CC8 11%, CC30 8%, CC45 17%). Five clusters contained only one genotype each. MRSA were distributed into 36 genotypes, MSSA into 81 genotypes whereas 3 genotypes were assigned to both MRSA and MSSA strains.

### *Spa *typing

The *spa *repeats were sequenced in 61 selected isolates on the basis of their distribution into the different clusters and of their polymorphism within these clusters. The sequence was submitted to the Ridom Spaserver in order to identify the *spa *type. Seven new *spa *types were given a number by the Ridom Spaserver. The result confirmed the correct clustering of strains by MLVA, as shown by the almost perfect correlation between the two genotyping techniques (Figures 2 and 3). Strain TrSa109 was positioned near CC30 strains and its *spa *type was characteristic of ST34 strains (CC30 members) a bacterial population resulting from a large chromosomal rearrangement between CC30 and CC8 [24]. Three identical isolates from patient CFU_79 which *spa *type corresponded to CC8 were branched in an ancestral position to the CC8 cluster.

Interestingly, in CC45 a larger diversity was observed for *spa *(10 alleles from 2 to 14 repeats), as compared to the other large clusters, CC5 (5 alleles) and CC8 (2 alleles).

### Longitudinal survey

In 62 patients (80%), isolates that were repeatedly recovered over the 30 months study period belonged to the same lineage, *i.e*. they were either identical or differed at only one VNTR. In the other patients the isolates belonged to 2, 3 or even 4 different CCs. For example isolates belonging to 4 different CCs were found in patient CFU_64 and only one of them was observed more than once. Table 2 shows the number of CCs and genotypes from patients for which at least 4 isolates were recovered. One example of stability is observed in patient CFU_41 for which 16 MOD-SA CC5 isolates with the same genotype were recovered from January 2006 to July 2008. Patient CFU_40 had 9 isolates with two different *spa *alleles. In 2006 and early 2007, a single genotype with seven repeats at the *spa *locus was observed, whereas after March 2007, isolates with two repeats at the *spa *locus were also found. On several occasions, both were present in equal amounts giving rise to two products upon PCR amplification (data not shown). From March 2006 to January 2008, 16 CC5 isolates were recovered from patient CFU_48, with three variants differing at VNTRs Sa1213 and Sa1132: one genotype was found in 7 isolates in 2006 and early 2007, another one in 8 isolates from 2006 to 2008 and the third corresponded to a single isolate in 2007. Patient CFU_96 had 14 isolates with three variants differing at VNTR Sa1132: one was found only in 2006 in two isolates, a second one was found once in 2006, 6 times in 2007 and once in 2008, and the third one was found twice in 2007. Patient CFU_34 had 7 isolates with two variants differing at VNTR Sa2039: one genotype was observed in 2006 in two isolates, and the other was found in five isolates once in 2006 and in 2007.

**Table 2:** Summary of the longitudinal survey in 24 patients with 4 or more isolates

Patient	isolates N°	First strain	cluster N°	genotype N°	variants^a^	CC
CFU_29	4	04/01/2006	1	1		15
CFU_25	7	05/03/2006	1	1		8
CFU_41	12	11/01/2006	1	1		5
CFU_36	13	21/01/2006	1	1		8
CFU_60	4	01/02/2006	1	1		8
CFU_76	4	26/04/2006	1	1		30
CFU_34	7	21/02/2006	1	1		30
CFU_59	8	04/01/2006	1	2	1866 (3; 2)	1
CFU_40	9	28/03/2006	1	2	122 (7; 2)	45
CFU_51	11	07/02/2006	1	2	906 (0.4; 0.3)	45
CFU_68	6	20/03/2006	1	3	0311 (5.5; 3.5), 1866 (3; 2)	45
CFU_22	7	21/02/2006	1	2	1425 (4; 1)	5
CFU_96	14	30/01/2006	1	3	1132 (4; 5; 6)	5
CFU_48	16	04/01/2006	1	3	1213 (5; 4), 1132 (6; 5)	5
CFU_63	4	02/02/2006	2	2		5(1), UN1^b^(3)
CFU_81	6	01/02/2006	2	2		8(2), 5(4)
CFU_82	6	03/02/2006	2	3	1756 (4;2)	30(2), 45(4)
CFU_97	6	03/01/2006	2	2		59(1), 45(5)
CFU_62	7	07/03/2006	2	2		5(5), 51(2)
CFU_11	6	18/01/2006	2	4	0122 (5; 4), 1729 (5; 3)	8(1), 45(5)
CFU_05	9	04/01/2006	3	3		7(1), 45(1), 5(7)
CFU_64	6	17/01/2006	4	4		30(3), 1(1), 51(1), UN2^c^(1)
CFU_26	12	19/01/2006	4	4		15(5), 45(3), 5(1), 7(3)

^a ^indicates the loci where there are VNTR variants within identical CC. In parentheses are shown the number of repeats at the variants.

^b ^UN1 corresponds to ST109

^c ^UN2 corresponds to ST398

### Genotypes and MRSA

On figures 2 and 3 are shown the sensitivity to methicillin and the presence/absence of the *mecA *gene carried by staphylococcal cassette chromosome *mec *(SCC*mec*), as tested by PCR. The large majority of MRSA isolates fall inside CC8, CC45 and CC5. In CC30, all strains were MSSA except for TrSa109 which is placed outside of the cluster and is *mec*A negative. Interestingly, in patient CFU_51, 10 isolates were of the same genotype, of which 6 were *mec*A positive and 4 were *mec*A negative, suggesting a recent transfer of the *mec*A gene or SCC*mec *instability in this particular strain. In five patients, isolates with identical genotypes were apparently either resistant or sensitive to methicillin but *mec*A was not detected while the phenotypic resistance aspects were BOR-SA or MOD-SA. In four patients only MSSA strains were isolated over more than 12 months (for example, in patient CFU_59 the same MSSA strain was isolated 7 times over 18 months).

The genetic diversity among MSSA isolates was larger than among MRSA, but both could be found in large CCs.

## Discussion

### Mlva

The MLVA procedure used in the present study allowed the systematic investigation of all *S. aureus *isolates recovered from CF patients attending a French centre during a period of 30 months. In the present study a total of 278 isolates from 79 children were genotyped, with a great variation within the number of *S. aureus *isolates per patient: one or two isolates for the firstly colonized patients (the youngest children), to more than 15 isolates for some chronically colonized patients repeatedly hospitalized for acute exacerbation. This type of study had never been conducted before because the available techniques were either time consuming or too expensive. Generally only MRSA isolates were studied and consequently, the MSSA diversity was insufficiently known although they account for a large proportion of strains responsible for chronic colonization in CF patients.

MLVA using 14 VNTRs is a very informative technique which compares favourably with MLST and *spa *typing. More genotypes are observed and it is possible to see the emergence of variants. The size of the VNTRs repeats ranges from 24 bp (the *spa *VNTR Sa0122) to 159 bp, which makes the technique very easy to implement using agarose gel electrophoresis as well as high throughput approaches. The allelic size differences for such markers can be estimated directly by eye and compared to a chart where all the known alleles have been indicated. This information is accessible on a dedicated web page in "The bacterial MLVA-genotyping-on-the-Web service" (http://mlva.u-psud.fr/; Staphylococcus aureus2009 database or a more recent update). For epidemiology purposes, a simpler scheme could be performed with a selection of 10 informative markers (MLVA-10). However, it is important to keep a large collection of markers with different degrees of variability for the investigation of outbreaks or for phylogenetic studies. In the present work each VNTR was amplified in a separate PCR reaction but our preliminary experiments showed that 6 VNTRs could be amplified simultaneously and the size automatically determined using a capillary electrophoresis apparatus [21]. This opens the way to automatized genotyping similarly to the protocol described by Schouls *et al*. [20]. However in this latest study only 8 VNTRs (MLVA-8) were analysed which, in our opinion may not be sufficiently discriminant for epidemiological studies. Indeed the Simpson's diversity index (DI) in the MLVA-8 assay was 98.5% whereas we obtained a 99.65% DI using the MLVA-14 assay. Other published VNTR-based genotyping methods either did not use enough markers or analyzed fingerprints which makes the comparison of profiles between laboratories very difficult [16]. In addition failure to amplify some VNTRs in a relatively important number of samples led to partial profiles in up to 27% of isolates in one study [19].

### Genetic diversity of strains and population structure

In the present collection of isolates, 110 genotypes were observed (not including the reference strains), 68% belonging to 4 main clusters. The genotypes in the MLVA cluster corresponding to CC8 were very stable over a period of more than 2 years. In contrast, more variability was observed in isolates of CC5 and CC45. In CC45, several VNTRs showed very small alleles as compared to the other clonal complexes which could be the result of frequent loss of repeats due to recombination.

The majority of CC8 isolates were MRSA, whereas these represented about 50% of CC5 and CC45 isolates. Recent studies have shown that CC8 contained both community and hospital-acquired MRSA strains whereas the other CCs mainly contained hospital-acquired strains [8,11,25]. In a recent survey of CF patients in Spain, 67% of MRSA isolates were ST228 which belong to CC5, and the second largest group corresponded to ST247 belonging to CC8 [26].

### Colonization

The precise identification of *S. aureus *genotypes colonizing the lungs of CF patients is of importance to trace the source of contamination and eventually modify the management of patients in the hospital. It is also important to know whether a single strain is present over time despite antibiotic treatment, or if different strains are involved in patients exacerbations. In two patients, isolates belonging to four different CCs were observed, but the most frequent situation was colonization by a single strain which could vary over time.

In some patients, the same strain was recovered over a two years period, with a few variants differing at a single VNTR. In several cases the variants seemed to appear sequentially suggesting that they acquired an advantage over the first isolate. On the contrary, in patient CFU_48, two variants were alternatively isolated during the two years period, and in patient CFU_40, the presence of two *spa *amplicons in a single PCR reaction pointed to the coexistence in equal amounts of two variants in the sputum sample. Interestingly variants were more frequently observed in CC45 strains than in other CCs, again indicating the existence of a higher degree of instability in this CC. It was shown that the adaptation to chronic colonization requires the expression of virulence factors and a higher mutation capacity resulting in an increase of the genetic diversity [27].

In 6 cases, a given genotype was shared by different patients, but it is difficult to define the origin of the contamination as most of these strains belonged to common CCs. Indeed, in a recent study by Sakwinska *et al*., it was shown that CC45 and CC30 colonized each 24% of the carrier population [28]. In the Armand Trousseau center, the risk of *P. aeruginosa *cross-colonization has led to the increased use of isolation protocols among the patients since many years. The source of *S. aureus *lung colonization could be either the nose, or the oro-pharynx, as suggested by recent studies [29,30]. The simplicity of MLVA genotyping should allow a systematic analysis of the first oro-pharyngal or nasal isolates of young CF patients and those chronically found in purulent sputum, as this may contribute to an early diagnosis of *S. aureus *infection. However searching for potential sources of *S. aureus *from the patients and their family members, the medical staff, the environmental home and hospital setting requires a laborious sampling work and needs another study.

Thirty eight patients were also colonized by *P. aeruginosa *as observed in a previous investigation [22]. There was apparently no link between the *S. aureus *genotype and the presence of *P. aeruginosa*. However, the patients from whom we analyzed a large number of *S. aureus *isolates, reflecting a long-term colonization, were usually coinfected with *P. aeruginosa*, with the exception of patient CFU_96 (14 isolates).

In a few patients, chronic colonization by a single strain was not observed although strains from up to 4 different CCs could be isolated during the study period.

### Antibiotic resistance

MRSA were found in more than 30% of patients, while some of them also carried MSSA. The presence of MRSA can limit the inscription of a patient on a lung transplant list [31], therefore, it is important to investigate the status and mechanisms of methicillin resistance. In some MRSA strains methicillin resistance was not associated with presence of *mec*A [32] and the resistance phenotype for most of these strains was BOR-SA, with overproduction of β-lactamase. Vancomycin was frequently used to treat MRSA infection, though pulmonary diffusion of this drug was not excellent. Eradication of *S. aureus *was rarely observed and chronic colonization was confirmed from repetitive sputum samples over time.

## Conclusion

In the present study, using the MLVA-14 procedure, we genotyped rapidly and with a simple equipment a large number of *S. aureus *isolates, allowing the longitudinal survey of 79 CF patients. A large proportion of isolates belonged to a limited number of CCs, and in most cases a single strain, either a MRSA or a MSSA, chronically colonized the patient. Over time variants appeared and it will be interesting to test whether they show selective advantages. The performances of MLVA open the way to additional studies to investigate the contamination sources and to identify *S. aureus *isolates responsible for colonization and clinical exacerbations.

## Methods

### Patients and bacterial strains

The criteria for diagnosis of CF was either the presence of 2 mutations in the *cftr *gene, or one or no mutation of *cftr *associated with a positive sweat test defined by a chloride (Cl^-^) ion concentration above 60 mmol/l. Sputum samples were collected from the lower airways, during an outpatient visit or hospitalization. For each patient an isolate was analysed with at least a one-month interval between two samples. A total of 278 isolates were genotyped from 79 patients (2 to 21 years old) attending the CF centre during the course of this study (January 2006 to June 2008). Patients were named CFU_ (for cystic fibrosis unit) as reported in a previous study on *P. aeruginosa *infection [22] and clinical isolates were named TrSa. The MLVA genotypes of the reference strains N315, USA300, MSSA 476, RF122, COL, NCTC8325, MRSA252, Mu50, MW2, JH1, JH9 and Newman were deduced from their genomic sequence by taking advantage of the tools available at http://minisatellites.u-psud.fr/. As a control for VNTR amplification, we used Mu50, an MRSA strain which genome has been sequenced [33]. The present project is in compliance with the Helsinki Declaration (Ethical Principles for Medical Research Involving Human Subjects). Strains were collected from sputum specimen as part of the patients' usual care, without any additional sampling. All patient data shown in the present work were anonymously reported, without offering any possibility to trace the actual patients. The "Comité Consultatif pour la Protection des Personnes dans la Recherche Biomédicale (CCPPRB) Ile-De-France - Paris - Saint Antoine" was consulted on April 4^th ^2006 and allowed the exemption of patient's written informed consent.

### Microbiology

Sputum samples were inoculated onto sheep blood, chocolate and salt mannitol agar (bioMérieux, France). After 2 days of incubation at 35°C, non identical-looking colonies were picked and tested for Gram stain and catalase reaction. Identification of *S. aureus *species was confirmed with the Pastorex Staph-Plus^® ^slide test (Bio-Rad, France), and antimicrobial susceptibility performed by disk diffusion. Methicillin (oxacillin) resistance was screened with the cefoxitin disk diffusion method, and the PBP 2a was detected with the MRSA-screen latex agglutination test (Denka, Seiken Co, Ltd, Japan) [34]. For MRSA isolates showing a negative PBP 2a agglutination test, overproduction of β-lactamase was screened looking for irregular aspect of the inhibition zone around the oxacillin disk, combined with a synergistic aspect between the inhibition zones of oxacillin and amoxicillin+clavulanate disks. The isolates with overproduction of β-lactamase are named BOR-SA (borderline *S. aureus*). In addition, detection of PBP 2a was performed in every MSSA isolates recovered from patients known to be previously colonized with MRSA.

### *mec*A gene detection

The presence of the *mec*A gene was searched by PCR amplification in all the isolates. Primers MecA_F, AAAATCGATGGTAAAGGTTGGC and MecA_R, AGTTCTGCAGTACCGGATTTGC were as described by Murakami *et al*. [35].

### DNA purification

About 20 colonies from a subculture on solid media were dissociated in 180 μl TE buffer (Tris 10 mM, EDTA 1 mM pH8). Then 20 μl of Lysostaphin (AMBICIN^® ^L, AMBI PRODUCTS LLC, Lawrence, USA) at a concentration of 1 mg/ml was added, the mixture was vortexed and incubated for 30 minutes at 37°C. One μl of Proteinase K (20 mg/ml) and 200 μl 2 × lysis Buffer (1% de SDS, 20 mM Nacl, 20 mM Tris pH8, 20 mM EDTA) were added, and the samples were incubated 30 minutes at 50°C. DNA was purified using phenol extraction and precipitation with ethanol. The quality and concentration of DNA was measured using a ND-1000 Spectrophotometer (NanoDrop^®^, Labtech, Palaiseau, France). The DNA was diluted at 1 ng/μl in water for the PCR amplification reaction.

### Genotyping

The MLVA-14 scheme is described in detail elsewhere [21], as well as its performance as compared to MLST and *spa *typing. In brief, it consists in the individual analysis of 14 VNTRs, five of which are located inside open reading frames (*spa*, *coa*, SIRU15, SAV1078, SAV 1738), six are in STAR elements and three are intergenic. Oligonucleotide primers targeting the 5' and 3' flanking regions of VNTR loci were used for amplification (Table 1). The following conditions were used: PCR reactions were performed in 15 μl containing 2 ng DNA, 1 × PCR Reaction Buffer, 1.5 mM MgCl_2_, 1 Unit of *Taq *DNA polymerase (Qiagen, Courtaboeuf, France), 200 μM of each dNTP, 0.3 μM of each flanking primer (Eurogentec, Angers, France). Amplification was performed with a PTC 200 thermocycler (Biorad, Marnes-la-Coquette, France) using the following conditions: initial denaturation cycle for 5 min at 94°C, 35 cycles of denaturation for 30 s at 94°C, annealing for 30 s at 58°C and elongation for 45 s at 72°C plus a final elongation step for 10 min at 72°C. For the analysis of all markers, 3 μl of PCR products were separated in a 2% agarose gel using agarose for routine use (Eurogentec, Angers, France). Electrophoresis was performed in 20 cm-wide gels made in 0.5 × TBE buffer (Sigma), run at 8 V/cm. For each PCR run the reference strain Mu50 was included. The 100-bp ladder DNA size marker was from MBI Fermentas (Euromedex, Souffelweyersheim, France). The gels were stained after the run in 0.5-1.0 g/ml ethidium bromide for 15 to 30 min, then rinsed with water and photographed under ultraviolet illumination (Vilber-Lourmat, Marne la Vallée, France). The amplicon size was determined using the dedicated tool Gelcompar in the BioNumerics software (Applied Math, Sint-Martens-Latem, Belgium).

The MLVA genotype of an isolate with 14 VNTRs (MLVA-14) is expressed as its allelic profile corresponding to the number of repeats at each VNTR in the order Sa0122 (alias *spa*), Sa0266 (alias *coa*), Sa0311, Sa0704, Sa1132, Sa1194, Sa1291 (alias SIRU13), Sa1729, Sa1866, Sa2039, Sa0906, Sa1213, Sa1425 and Sa1756 (alias SIRU15). The genotype of the Mu50 strain deduced from its genomic sequence is 10 6 3 4 6 7 4 5 3 3 3 5 4 1. Clustering analyses were performed using the categorical coefficient (also called Hamming's distance) and UPGMA. A cut off value of 45% was previously shown [21] to define clusters which correspond to MLST clonal complexes and is therefore used in this study to identify CCs. The isolates in these CCs differ at a maximum of three VNTRs out of 14. Lineages are arbitrarily defined as groups of isolates for which the genotype between two isolates differs at a maximum of 2 VNTRs (cut-off 80%). The minimum spanning tree was produced in BioNumerics, scaling with member count. The polymorphism index of individual or combined VNTR loci was calculated using the Hunter-Gaston diversity index (*HGDI*) [36], an application of the Simpson' s index of diversity [37] is 0.9965 [21].

### *Spa *typing

The *spa *tandem repeat was amplified using the primers for Sa0122, and the amplicons were purified by polyethylene glycol (PEG) precipitation and sequenced (MWG Biotech). The repeat nomenclature was that of [14], and *spa *type were obtained from the spa typing website http://www.spaserver.ridom.de/ developed by Ridom GmbH and curated by SeqNet.org http://www.SeqNet.org/ [38]. The *spa *types were correlated to the MLST CCs according to the SpaServer.

### MLST typing

The primers and condition used for PCR were found on the mlst.net at http://saureus.mlst.net/.

## Authors' contributions

BF and HC participated in the design of the study and provided clinical samples and information. DM carried out bacterial culture and identification. KH and GC carried out the molecular genetic studies. GV participated in the design of the study and performed bioinformatics analysis. HVT and CP conceived of the study, and participated in its design and coordination and drafted the manuscript. All authors read and approved the final manuscript.
